# Evaluation of Untargeted Metabolomic Strategy for the Discovery of Biomarker of Breast Cancer

**DOI:** 10.3389/fphar.2022.894099

**Published:** 2022-05-30

**Authors:** Xujun Ruan, Yan Wang, Lirong Zhou, Qiuling Zheng, Haiping Hao, Dandan He

**Affiliations:** ^1^ Key Laboratory of Drug Metabolism and Pharmacokinetics, State Key Laboratory of Natural Medicines, China Pharmaceutical University, Nanjing, China; ^2^ Department of Pharmaceutical Analysis, College of Pharmacy, China Pharmaceutical University, Nanjing, China; ^3^ Experimental Center of Molecular and Cellular Biology, The Public Laboratory Platform, China Pharmaceutical University, Nanjing, China

**Keywords:** untargeted metabolomics, strategy evaluation, biomarker discovery, breast cancer, UPLC-MS

## Abstract

Discovery of disease biomarker based on untargeted metabolomics is informative for pathological mechanism studies and facilitates disease early diagnosis. Numerous of metabolomic strategies emerge due to different sample properties or experimental purposes, thus, methodological evaluation before sample analysis is essential and necessary. In this study, sample preparation, data processing procedure and metabolite identification strategy were assessed aiming at the discovery of biomarker of breast cancer. First, metabolite extraction by different solvents, as well as the necessity of vacuum-dried and re-dissolution, was investigated. The extraction efficiency was assessed based on the number of eligible components (components with MS/MS data acquired), which was more reasonable for metabolite identification. In addition, a simplified data processing procedure was proposed involving the OPLS-DA, primary screening for eligible components, and secondary screening with constraints including VIP, fold change and *p* value. Such procedure ensured that only differential candidates were subjected to data interpretation, which greatly reduced the data volume for database search and improved analysis efficiency. Furthermore, metabolite identification and annotation confidence were enhanced by comprehensive consideration of mass and MS/MS errors, isotope similarity, fragmentation match, and biological source confirmation. On this basis, the optimized strategy was applied for the analysis of serum samples of breast cancer, according to which the discovery of differential metabolites highly encouraged the independent biomarkers/indicators used for disease diagnosis and chemotherapy evaluation clinically. Therefore, the optimized strategy simplified the process of differential metabolite exploration, which laid a foundation for biomarker discovery and studies of disease mechanism.

## Introduction

Metabolites have been realized to play an important role in the onset of diseases, and are of great significance for disease diagnosis and prevention. Metabolomics is attracting increasing attentions in various areas, such as pathological mechanism studies, pathway analysis, and the exploration of novel biomarkers for diseases, including cancers (Armitage and Southam, 2016; Kumar and Misra, 2019; Liu et al., 2021; Long et al., 2021). According to different research goals, there comes up with untargeted metabolomics and targeted metabolomics (Jasbi et al., 2019; Casari et al., 2021; Baek et al., 2022; Harrieder et al., 2022). Targeted metabolomics concentrates more on the analysis of defined metabolites, which has limited scope but achieves enhanced detection sensitivity and enables the absolute quantification with the application of standards (Roberts et al., 2012; Cai et al., 2015; Zhou and Yin, 2016). Untargeted metabolomics, on the other hand, has superiority in high-throughput detection, which offers a comprehensive and in-depth insight of metabolome profiling (Yuan et al., 2012; Heiles, 2021) and is regarded as the basis for biomarker discovery.

Mass spectrometry (MS) has been regarded as a powerful analytical technique owing to its high detection speed and sensitivity. Its coupling with chromatographic separation, such as gas chromatography and liquid chromatography (LC), has been widely used for metabolomic analysis by providing both molecular weight and structural information (Liu et al., 2013; Alonso et al., 2015; Nash and Dunn, 2019; Hou et al., 2020; Harrieder et al., 2022). Efforts have been done in method development for sample preparation, chromatographic separation and derivatization-based detection to improve the metabolome coverage (Yuan et al., 2018; An et al., 2021; Meng et al., 2021). Besides, computational approaches are also dramatically developed to assist data interpretation and metabolite global annotation (Bonini et al., 2020; Chen et al., 2021; Duehrkop et al., 2021).

Breast cancer has become one of the leading causes threating health in women and its incidence is increasing within recent years. With the development of new therapeutic strategies, the mortality of breast cancer has gradually reduced (DeSantis et al., 2019). Metabolomics has been widely applied for metabolic pathway analysis and biomarker discovery for breast cancer based on the analysis of different biological samples, including cell lines, plasma, serum, tissues, urine and saliva (Tsutsui et al., 2013; Tenori et al., 2015; Zhong et al., 2016; Porto-Figueira et al., 2018), which facilitates the early diagnosis, treatment target exploration and mechanism studies of the disease (Günther, 2015; McCartney et al., 2018; Park et al., 2019; Silva et al., 2019; Long et al., 2021). Methodologies with various sample preparation steps, detection methods, data processing and metabolite annotation procedures were developed, upon which the evaluation is essential before sample analysis due to the difference of sample property or experimental purpose. Herein, sample preparation, data processing procedure, and metabolite identification strategy of untargeted metabolomics were evaluated and subsequently applied for the analysis of serum samples of breast cancer. First, metabolite extraction by different solvents was assessed and evaluation based on the number of components with MS/MS data acquired (defined as eligible components) was regarded to be more reasonable in consideration of metabolite identification. In addition, a simplified data processing procedure was proposed involving orthogonal projections to latent structures discriminant analysis (OPLS-DA) for all detected components, followed up with a primary screening based on the availability of MS/MS data and underwent a secondary screening with criteria of VIP, fold change (FC) and *p* value. Thus, only differential candidates were subjected to database search, identification and annotation, which greatly reduced the data volume and improved the analysis efficiency. Furthermore, the confidence and accuracy of metabolite identification were enhanced by comprehensively considering mass and MS/MS errors, isotope similarity, fragmentation match and biological source confirmation. On this basis, the evaluated strategy was applied for the analysis of serum samples of breast cancer, upon which the discovery of potential biomarkers would be informative for early diagnosis and chemotherapeutic evaluation of the disease.

## Materials and Methods

### Chemicals and Reagents

Formic acid (FA) was purchased from Sigma-Aldrich (Saint Louis, MO, United States). Ultra-pure H_2_O was prepared by a Milli-Q Pure Water System (Bedford, MA, United States). LC-MS grade methanol (MeOH) and acetonitrile (ACN) were purchased from Merck (Darmstadt, Germany).

### Clinical Sample Collection and Sample Preparation

Serum samples were collected from Huashan Hospital, Fudan University and stored at −80°C before analysis. The research protocol was approved by the Ethical Committee of Huashan Hospital, Fudan University (KY2021-034), and written informed consents were provided by all participants.

For sample preparation, 400 μl of designed solvent (pre-cooled on ice) was mixed with 100 μl of serum sample and followed up with a 2 min vortex for sufficient extraction and protein precipitation. The obtained mixture was centrifuged at 15,000 g (5 min at 4°C) and directly stored at 4°C (1 h) to ensure the complete protein precipitation. Supernatant (200 μl) was transferred for an additional centrifugation (18,000 g for 5 min at 4°C). Vacuum-dried and re-dissolution by extraction solvent was performed prior to MS analysis if necessary. Quality control (QC) sample was prepared by pooling aliquots of each serum sample investigated in this study, including 58 samples from breast cancer vs. healthy control and 12 samples from breast cancer patients before and after chemotherapy. The prepared QC sample was applied for extraction evaluation and instrumental performance monitoring.

### UPLC-MS

Chromatographic separation was performed by a Waters ACQUITY I-Class UPLC system equipped with an ACQUITY UPLC HSS T3 column (2.1 × 100 mm, 1.8 μm, Waters). Parameters were set as follows: column temperature 40°C; flow rate of 0.4 ml/min; injection volume of 4 μl; mobile phase A was H_2_O containing 0.1% FA and B was ACN. The gradient elution condition was referenced by previous study (He et al., 2021): 0–1.00 min, 0% B; 1.01–4.00 min, 0–35% B; 4.01–15.50 min, 35–95% B; 15.51–18.00 min, maintaining at 95% B; 18.01–23.00 min, back to 0% B. MS detection and data acquisition were performed by a Q-TOF MS (Waters, Xevo, G2-XS QTof) in both positive and negative ion modes. MS parameters were set as follows: *m/z* range at 50–700 Da; capillary voltage at + 3.0 kV or −2.5 kV for positive or negative ion mode respectively; sampling cone at 40 V; source temperature at 110°C; desolvation temperature at 450°C; cone gas at 50 L/h, desolvation gas at 600 L/h. MassLynx (version 4.1, Waters) was used for data acquisition.

### Data Processing

Data processing was performed by Progenesis QI (version 2.0, Waters; denoted as QI), which mainly contained steps of: creation of a new experiment; data import; review alignment; experiment design setup; peak picking; review deconvolution; compounds identification; review compounds; and compound statistics. Notably, adduct ion forms, such as [M + H]^+^, [M-H]^−^, [M + Na]^+^, [M + K]^+^, [2M-H]^−^, and [M + FA-H]^−^, were all included. Different adduct ion forms of a same metabolite were automatically combined to provide an accurate identity. For metabolites having different adduct ion forms, the one with the highest MS intensity was selected to represent the abundance. Databases for metabolite identification and annotation included Human Metabolome Database (HMDB) (http://www.hmdb.ca/), MoNA (http://mona.fiehnlab.ucdavis.edu/), and METLIN (https://metlin. scripps.edu/). Parameters were set as follows: 10 ppm for precursor ion match; 20 ppm for fragment ion match; > 80% for the isotope similarity; ≥ 40 score of identification. The MS/MS spectra for potential biomarkers were manually checked to confirm the assigned identities. Relative quantification of selected metabolites by QI was performed by integration of corresponding extracted ion chromatograms (EICs) and normalized against the total ion chromatogram (TIC). Manually check of EIC was required to ensure the accurate peak picking and correct integration.

### Statistical Analysis

Components detected were exported by QI and subjected to EZinfo software (version 3.0, Waters) for OPLS-DA for statistical difference confirmation. Components with variable importance calculated based on S-plots (VIP) > 1, *p* value <0.05 (student’s t-test) and FC > 1.5 were screened. Notably, for the same substance detected under different ion modes, the one with the smallest FC were adapted for further data analysis. Bioinformatics analysis of assigned differential metabolites, including pathway analysis and receiver operating characteristic (ROC) curve, were performed on MetaboAnalyst (version 5.0) (http://www.metaboanalyst.ca/).

## Results and Discussion

### Evaluation of Untargeted Metabolomics

**FIGURE 1:**
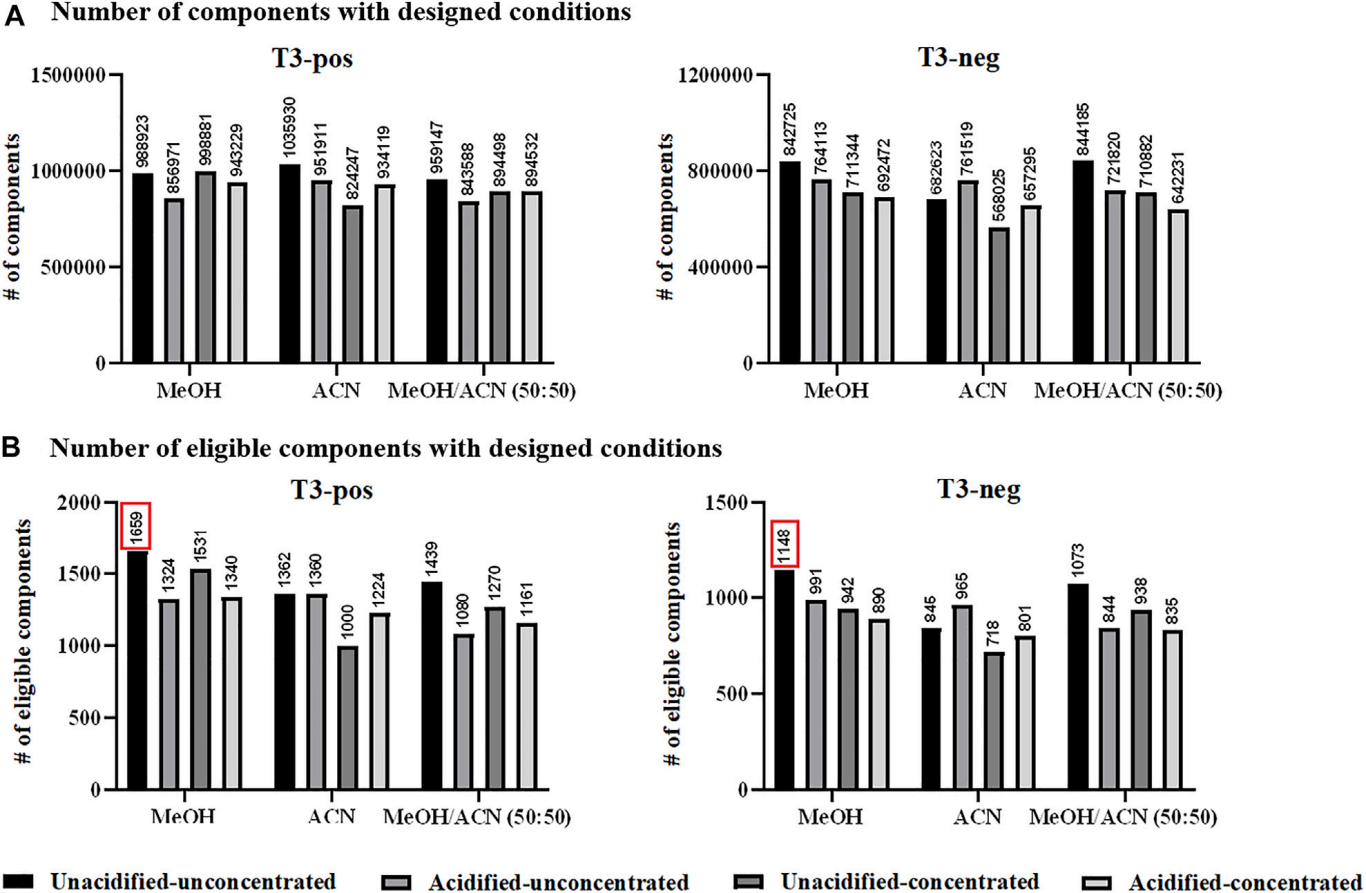
**(A)** Total number of components (considering MS1 data) detected with designed conditions; and **(B)** total number of eligible components detected with designed conditions. Pos: positive ion mode; neg: negative ion mode.

*Sample preparation.* Metabolite extraction and protein precipitation by two commonly used solvents, MeOH and ACN, as well as their mixture (50:50 by volume) were evaluated. The option of 0.1% FA adoption was also explored, as well as vacuum-dried concentration and re-dissolution considering metabolites with low abundance. The analysis of designed solvent-treated QC samples resulted to over 800,000 ions in the positive ion mode, among which ACN-extraction appeared to be the optimal one. In the negative ion mode, at least 500,000 ions were detected upon designed extraction conditions, and MeOH/ACN mixture had a higher efficiency (Figure 1A). Nevertheless, it was still hesitant to choose one appropriate solvent while such comparison was rough and inappropriate. Aiming at metabolite identification, evaluation based on eligible components was proposed to be more reasonable. Accordingly, eligible components obtained by designed conditions were in the range of 1,000–1,700 in the positive ion mode, among which MeOH-extraction was optimum with 1,659 components having MS/MS data. Similarly, extraction by designed solvents resulted to eligible components ranging from 700 to 1,200 in the negative ion mode, among which MeOH also emerged by having 1,148 components detected with MS/MS spectra (Figure 1B). Interestingly that no significant improvement was observed after concentration, which was probably due to the decomposition of unstable compounds during the tedious vacuum-dried step. Thus, MeOH extraction was applied for subsequent serum sample preparation and the obtained mixture after protein precipitation was directly subjected to MS analysis without further treatment. Moreover, the number of components varied with different extraction solvents further supported the importance of evaluation before sample analysis.

**FIGURE 2:**
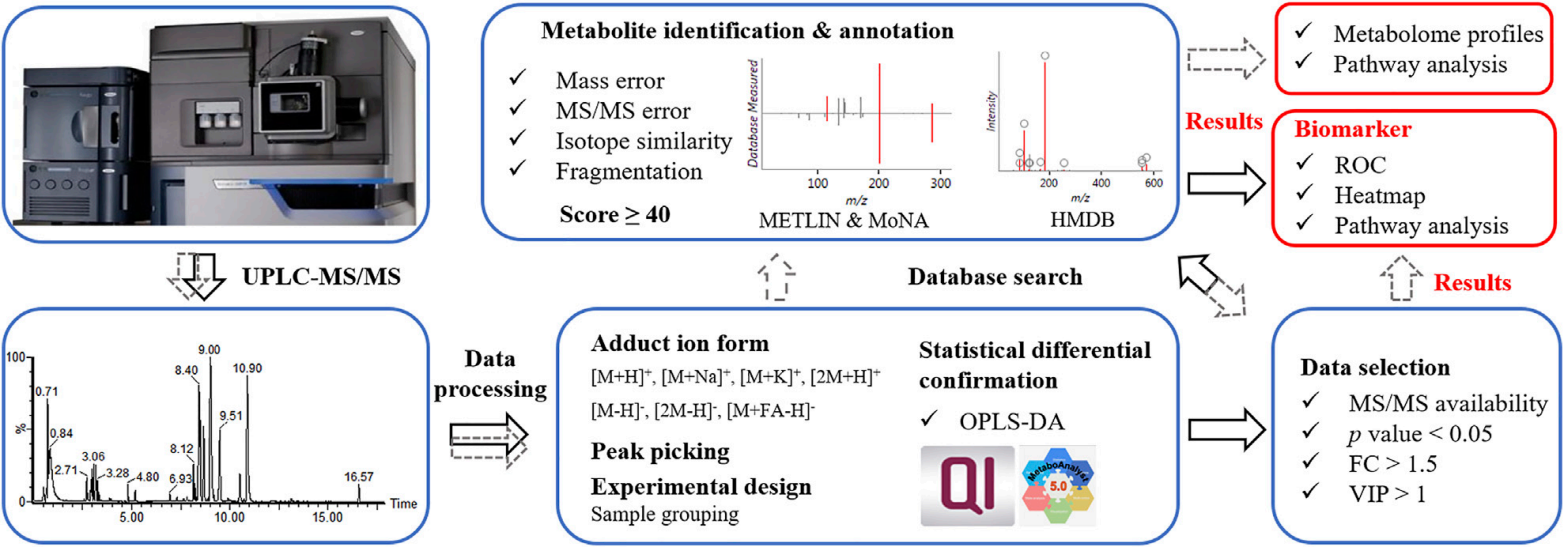
Data processing procedures. Grey dash line referred to a routine data processing procedure; black solid line referred to the proposed simplified procedure.

*Data processing procedure.* Upon UPLC-MS analysis, resulted components (considering MS1 data here) underwent OPLS-DA to ensure the statistical difference between tested groups. For routine procedure (Figure 2, in grey dash line), components with MS/MS data were subjected to database search, metabolite identification and differential analysis. Such procedure involved annotation and confirmation for every component, which was informative for revealing the difference in metabolome profiles and metabolomic pathways between tested samples. Aiming at the discovery of biomarker, we proposed a simplified data processing procedure with improved analysis efficiency. As shown in Figure 2 (in black solid line), OPLS-DA was applied for multivariate analysis. Primary screening was performed to select eligible components, among which differential ones were further isolated by secondary screening with criteria of *p* value <0.05, VIP >1 and FC > 1.5. Thus, instead of massive identification, only differential candidates were allowed to subsequent database search and identification, which greatly narrowed down the data volume for interpretation.

*Metabolite identification strategy.* For metabolite identification and annotation, parameters including *m/z* error, fragment error, isotope similarity, and fragmentation match were mainly concerned. Fragmentation match was performed based on databases including METLIN, MoNA and HMDB. The former two contained MS/MS spectra that experimentally collected, while HMDB offered theoretical fragmentation based on molecular structures. Metabolite identification was relied on the Score value from QI, which comprehensively evaluated the match degree of all above mentioned parameters. Based on previous study (Hou et al., 2020), the annotation accuracy and confidence were enhanced by setting a threshold of Score ≥40 for acceptance, which was higher than the one commonly used (≥35). Moreover, biological source information provided complementary information for further confirmation, upon which the structural and biological information was integrated for identification and annotation. Notably that it was possible to exclude a small portion of potential biomarkers based on such data processing procedure and identification strategy, especially for those with MS1 data only or those were not included in the databases we used. However, it was known that the identification of such components was challenge due to limited information provided, which would decrease the annotation confidence and reliability. Thus, such potential biomarkers would not be considered in this study as the identification and annotation with high confidence and accuracy were taken as the primary standard and purpose.

### Exploration of Potential Biomarker of Breast Cancer

**FIGURE 3:**
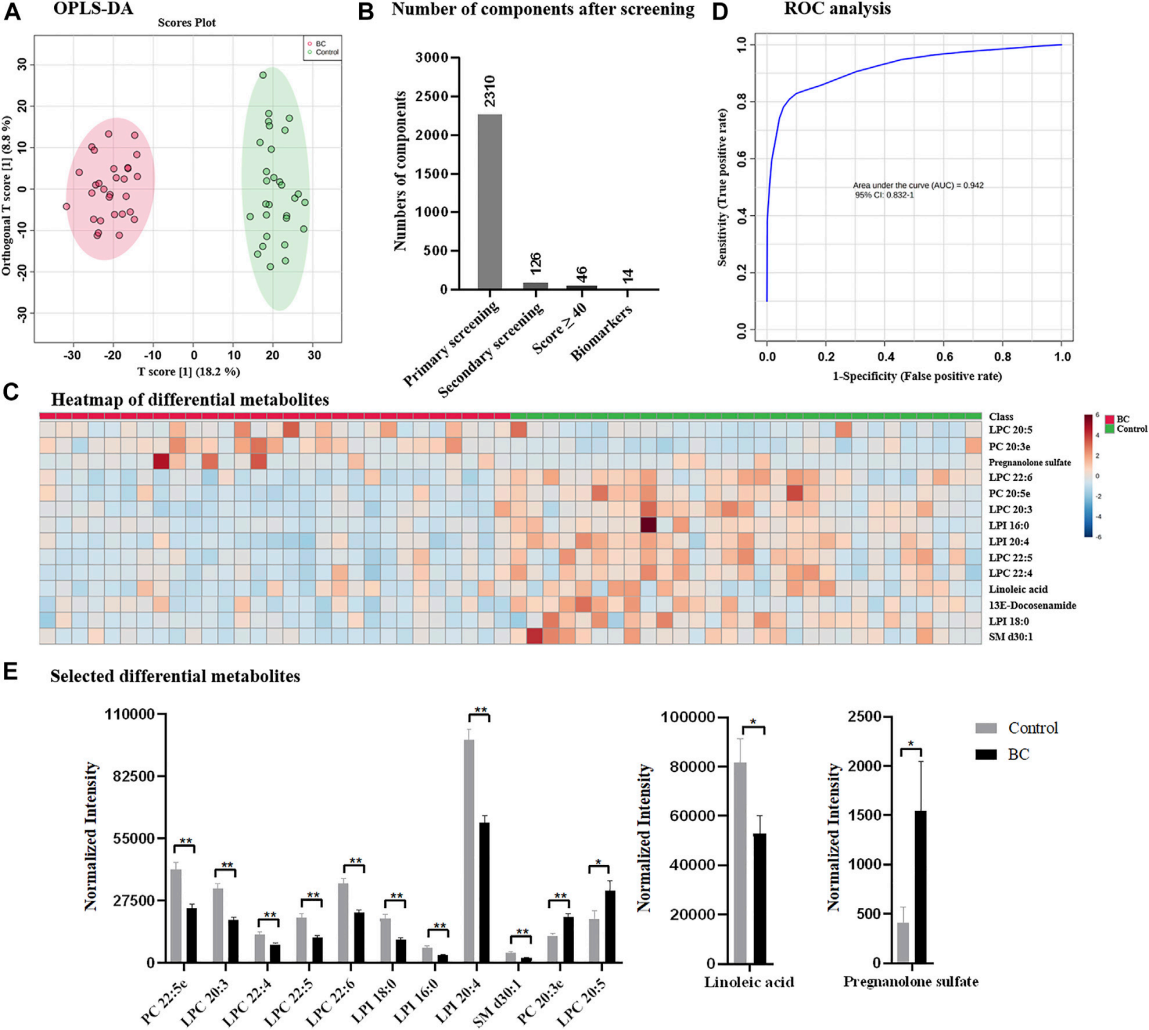
**(A)** OPLS-DA score plots derived from the serum metabolomics datasets collected from healthy control and breast cancer group; **(B)** number of components after designed screening steps; **(C)** heatmap of differential metabolites; **(D)** ROC analysis; and **(E)** selected differential metabolites between healthy control and breast cancer group. BC: breast cancer.

Following the optimized strategy, 58 serum samples containing 29 from healthy control and 29 from breast cancer patients were investigated. The instrumental stability and reproducibility were assessed by QC sample based on five successive injections prior to sample analysis and interval injections during sample run (Supplementary Figure S1A). Upon UPLC-MS analysis, total 8,399 components (considering MS1 data only) were detected in the positive and negative ion modes. According to OPLS-DA, healthy control and breast cancer group exhibited distinguish metabolome profiles and were completely separated with R^2^Y of 96.4% and Q^2^ of 83.3% (Figure 3A). Following the proposed simplified procedure, primary screening resulted to 2,310 eligible components with qualified MS/MS data and only 126 differential candidates were remained after secondary screening for subsequent database search (Figure 3B), which greatly reduced the data volume and saved efforts for data interpretation. The identification by QI further narrowed down the number of metabolites to 46 with Score value ≥40. Upon biological source confirmation, a total of 14 endogenous metabolites were finally emerged (details found in Supplementary Table S1; Supplementary Figure S2). Heatmap analysis in Figure 3C also confirmed that they had quantitatively difference between healthy control and breast cancer group, according to which a considerable number of metabolites were observed down-regulated in breast cancer group. ROC curve analysis was applied to verify the representative of annotated metabolites and AUC of 0.942 in Figure 3D confirmed the reliability of identified metabolites, which implied them as potential biomarkers for clinical diagnosis of breast cancer. Differential metabolites mainly belonged to lipids, fatty acid, and fatty amide, some of which were also reported previously (Long et al., 2021) (Figure 3E). Lipids accounted for the majority of differential metabolites, including two phosphorylcholines (PCs), five lysophosphatidylcholines (LPCs) and three lysophosphatidylinositol (LPIs), which were also reported to be closely related to the occurrence of breast cancer (Cala et al., 2018; Song et al., 2020; Long et al., 2021). Besides, linoleic acid, down-regulated in breast cancer group, was reported to be closely related to the regulation of breast cancer involved miRNA expression (Elieh Ali Komi et al., 2021). Pregnanolone sulfate, significantly up-regulated in breast cancer group based on our results, suggested a possible relationship between breast cancer and steroids. (Bicikova et al., 2001). It was worth to mention that the sample size enrolled in this study was limited, which might lead to insufficient discovery and miss other potential biomarkers. A larger sample population or detailed classification in subtypes, disease progression or treatment would be more expected.

**FIGURE 4:**
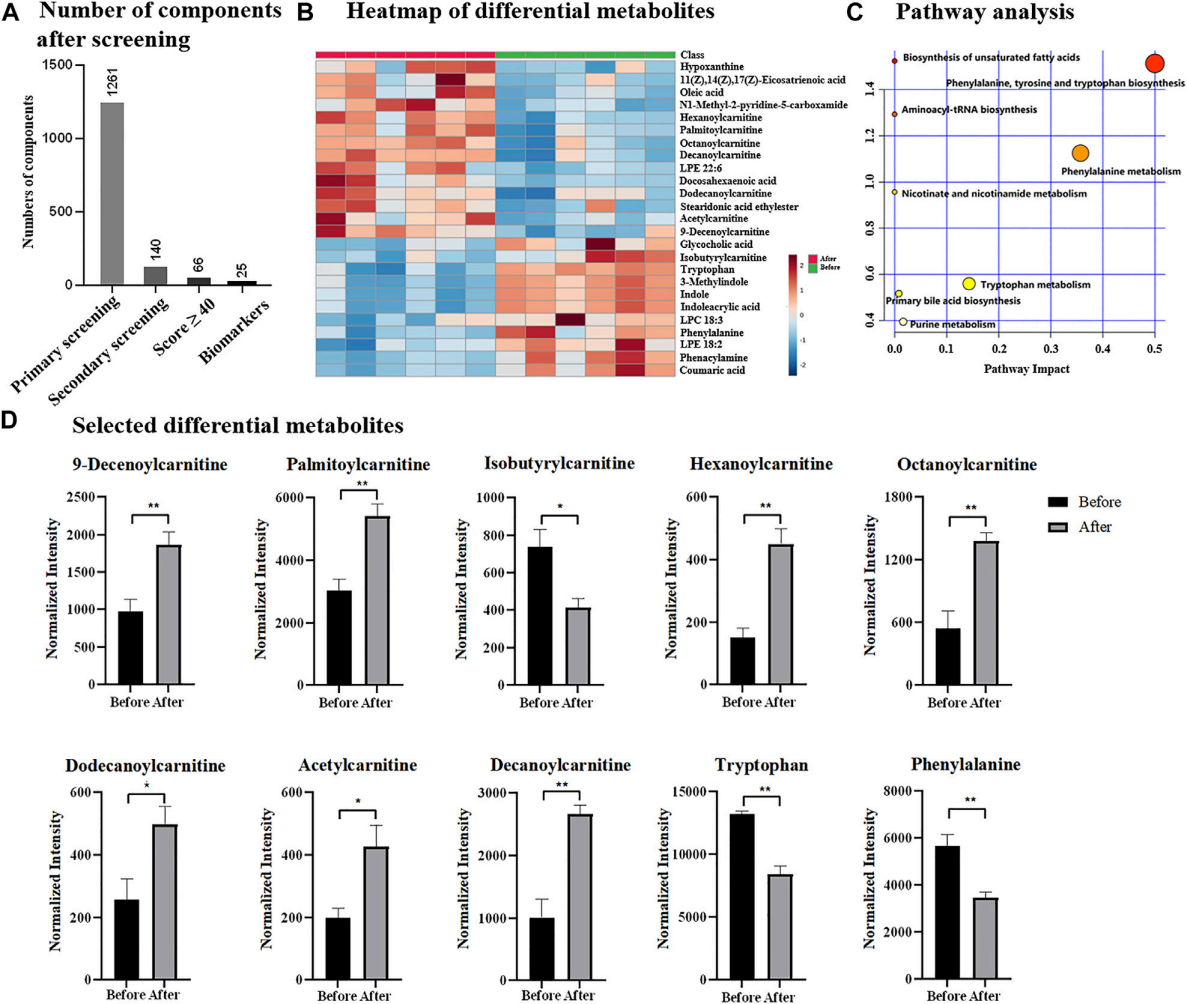
**(A)** Number of components after designed screening steps; **(B)** heatmap of differential metabolites; **(C)** pathway analysis; **(D)** selected differential metabolites before and after chemotherapy.

Chemotherapy is one of major treatments for breast cancer, thus, the discovery of potential indicators is also essential for monitoring the process or effect of chemotherapy. Herein, total 12 serum samples collected from six breast cancer patients were investigated, including six samples before chemotherapeutic treatment and six samples after chemotherapeutic treatment. Upon UPLC-MS analysis, total 6,130 components (considering MS1 data only) were detected and based OPLS-DA (Supplementary Figure S3A), two groups were statistically different by receiving a R^2^Y of 99.8% and Q^2^ of 77.8%. With the simplified data processing procedure, 1,261 eligible components with qualified MS/MS data were isolated, while 140 of them met the criteria of secondary screening and were remained as differential candidates for database search. Based on QI identification, 66 components received Score value ≥40, and 25 of them were finally emerged as potential biomarkers according to biological source confirmation (Figure 4A, details found in Supplementary Table S2 and Supplementary Figure S4). In addition, heatmap analysis illustrated significant quantitative difference among two groups, including 11 metabolites were down-regulated and 14 were up-regulated in chemotherapeutic group (Figure 4B and Supplementary Table S2). ROC analysis in Supplementary Figure S3B confirmed them as representative and potential indicators for monitoring the process or treatment of chemotherapy by achieving AUC of 1.00. Notably that the AUC value of 1.00 was not common, which was probably due to the indeed significant difference of potential metabolites before and after chemotherapeutic treatment. Besides, it was also because of limited sample size, which resulted to overfitting during ROC analysis. The identified potential indicators mainly distributed in biosynthesis of unsaturated fatty acids, aminoacyl-tRNA biosynthesis, nicotinate and nicotinamide metabolism, as well as phenylalanine, tyrosine and tryptophan biosynthesis (Figure 4C), some of which had also been reported previously as biomarkers for breast cancer diagnosis (Long et al., 2021). Among differential metabolites included eight carnitine species, suggesting the possible dysregulation of carnitine metabolism and fatty acid *β*-oxidation process. Phenylalanine and tryptophan were reported previously to be dysregulated and were highly expressed in breast cancer based on tissue or cell investigation (Du et al., 2019; Long et al., 2021). Similarly, indole was also considered as one of biomarkers for breast cancer and discovered to be up-regulated based on plasma analysis (Jasbi et al., 2019). They were observed down-regulated after chemotherapy according to results in Figure 4D and Supplementary Table S2, suggesting them as potential indicators to track chemotherapeutic treatment and disease recovery. Notably that differential metabolites identified in this study had overlap but not identical with previous studies, which could be attributed to the difference in sample types (tissue, serum or cultured cells). In addition, the differential metabolites identified for chemotherapeutic samples were different from that of breast cancer vs. healthy group, suggesting the possibility that the potential biomarkers for breast cancer diagnosis and chemotherapeutic monitoring were independent. It also implied that for clinical applications, the same potential biomarkers for breast cancer diagnosis were not sufficient for evaluation of chemotherapy, as separated metabolic pathways were probably affected. Thus, the discovery of indicators specified for the evaluation of chemotherapy were highly encouraged and large sample size would be more expected, especially for samples collected from the same subtype, progression of the disease or treatment.

## Conclusion

In this study, sample preparation, data processing procedure and metabolite identification strategy of untargeted metabolomics were evaluated. Metabolite extraction by different solvents was investigated and reasonable assessment based on the number of eligible components was proposed. A simplified data processing procedure was proposed, involving OPLS-DA for statistical difference confirmation, primary screening based on MS/MS data availability and secondary screening according to criteria including FC, VIP and *p* value. Such procedure allowed database search for differential components only, which greatly narrowed down the data volume, improved the analysis efficiency and facilitated to reduce false identification results. For identification and annotation, mass error, MS/MS error, isotope similarity and fragmentation match were comprehensively considered and Score ≥40 was set to enhance the identification confidence and accuracy. The evaluated strategy was applied for the analysis of serum samples of breast cancer and the discovery of distinguished metabolites highly encouraged the exploration of independent biomarkers for disease diagnosis and treatment clinically. Therefore, the evaluated strategy was beneficial for the discovery of potential biomarker with the simplified procedure and unambiguous annotation.

## Data Availability

The original contributions presented in the study are included in the article/Supplementary Material, further inquiries can be directed to the corresponding authors.
