# Supplementary material for: Evaluation of Untargeted Metabolomic Strategy for the Discovery of Biomarker of Breast Cancer
Source: Front Pharmacol. 2022 May 30;13:894099. doi: 10.3389/fphar.2022.894099 (PMC9189413; doi:10.3389/fphar.2022.894099)
Supplement: Supplementary file 1 [file Table1.pdf]

Supplementary Material

Evaluation of Untargeted Metabolomic Strategy for the Discovery of Biomarker of Breast Cancer

Xujun Ruan [a,1], Yan Wang [c,1], Lirong, Zhou [c], Qiuling Zheng [c] *, Haiping Hao [a] *, Dandan He [b] *

[a] Key Laboratory of Drug Metabolism and Pharmacokinetics, State Key Laboratory of Natural Medicines, China Pharmaceutical University, Tongjiaxiang #24, Nanjing, Jiangsu, 210009, China

[b] Experimental Center of Molecular and Cellular Biology, the Public Laboratory Platform, China Pharmaceutical University, Tongjiaxiang #24, Nanjing, Jiangsu, 210009, China

[c] Department of Pharmaceutical Analysis, College of Pharmacy, China Pharmaceutical University, Tongjiaxiang #24, Nanjing, Jiangsu 210009, China.

Table S1. Metabolites with significant difference between healthy control and breast cancer patients.

RT (min)	Metabolite	Formula	Mass (Da)	Detection mode	Adducts*	Observed (*m/z*)	Mass error (*ppm*)	Fold change	Up/down regulation	*P* value
7.62	LPC 20:5	C28H48NO7P	541.3186	Pos	M+H	542.3259	3.22	1.67	↑	2.32E-02
8.79	PC 20:3e	C28H52NO7P	545.3481	Pos	M+H	546.3577	4.12	1.70	↑	3.10E-06
16.57	13E-Docosenamide	C22H43NO	337.3345	Pos	M+H	338.3418	0.06	1.58	↓	1.08E-03
7.27	Pregnanolone sulfate	C21H34O5S	398.2127	Neg	M-H	397.2074	4.92	3.78	↑	3.59E-02
8.59	LPI 18:0	C27H53O12P	600.3275	Neg	M-H	599.3213	1.80	1.92	↓	1.76E-05
8.82	LPC 20:3	C28H52NO7P	545.3481	Neg	M+FA-H	590.3469	1.07	1.74	↓	1.78E-06
11.05	LPI 20:4	C29H49O12P	620.2962	Neg	M-H	619.2888	-0.08	1.59	↓	2.22E-08
12.54	LPI 16:0	C25H49O12P	572.2962	Neg	M-H	571.2928	6.78	1.95	↓	6.64E-04
13.48	Linoleic acid	C18H32O2	280.2427	Neg	M-H	279.2344	8.72	1.55	↓	2.08E-02
8.21/8.23	LPC 22:6	C30H50NO7P	567.3325	Pos/Neg	M+H/M+FA-H	568.3415/612.3304	3.01/-0.50	1.71/1.59	↓	1.90E-07/7.96E-08
8.87/8.90	PC 22:5e	C30H52NO7P	569.3481	Pos/Neg	M+H/M+FA-H	570.3574/614.3466	3.45/0.39	2.17/1.71	↓	9.68E-09/8.53E-06
9.32/9.34	LPC 22:5	C30H52NO7P	569.3481	Pos/Neg	M+H/M+FA-H	570.3578/614.3464	4.19/017	2.26/1.81	↓	1.58E-07/3.485-05
9.80/9.82	LPC 22:4	C30H54NO7P	571.3638	Pos/Neg	M+H/M+FA-H	572.3729/616.3617	3.28/-0.51	1.87/1.55	↓	7.18E-06/5.03E-04
14.78/14.83	SM d30:1	C35H71N2O6P	646.5050	Pos/Neg	M+H/M+FA-H	647.5177/691.5095	8.40/9.84	2.24/2.92	↓	7.07E-05/3.37E-03

* For metabolites detected in both positive and negative ion modes, the one with smallest FC was selected for further analysis.

Table S2. Metabolites with significant difference for breast cancer patients before and after chemotherapy.

RT (min)	Metabolite	Formula	Mass (Da)	Detection mode	Adducts*	Observed(*m/z*)	Mass error *(ppm)*	Fold change	Up/down Regulation	*p* value
0.74	Acetylcarnitine	C9H17NO4	203.1158	Pos	M+H	204.1230	0.00	2.15	↑	1.28E-02
1.82	Hypoxanthine	C5H4N4O	136.0385	Pos	M+H	137.0464	4.68	1.87	↑	1.51E-02
2.45	Phenacylamine	C8H9NO	135.0684	Pos	M+H	136.0763	4.15	1.56	↓	7.63E-04
2.46	Coumaric acid	C9H8O3	164.0473	Pos	M+H	165.0549	1.81	1.53	↓	8.69E-04
2.55	N1-Methyl-2-pyridone-5-carboxamide	C7H8N2O2	152.0586	Pos	M+H	153.0662	2.31	2.69	↑	2.44E-03
2.84	Isobutyryl-carnitine	C11H21NO4	231.1471	Pos	M+H	232.1544	0.41	1.78	↓	1.09E-02
2.98	Indole	C8H7N	117.0578	Pos	M+H	118.0657	4.96	2.05	↓	9.40E-06
2.98	3-Methylindole	C9H9N	131.0735	Pos	M+H	132.0810	1.50	1.82	↓	1.43E-05
2.98	Indoleacrylic acid	C11H9NO2	187.0633	Pos	M+H	188.0708	1.19	1.95	↓	1.15E-05
3.67	Hexanoylcarnitine	C13H25NO4	259.1784	Pos	M+H	260.1857	0.22	2.98	↑	2.99E-04
4.34	Octanoylcarnitine	C15H29NO4	287.2097	Pos	M+H	288.2171	0.56	2.53	↑	8.62E-04
4.67	9-Decenoylcarnitine	C17H31NO4	313.2253	Pos	M+H	314.2334	2.49	1.9	↑	3.70E-03
4.91	Decanoylcarnitine	C17H33NO4	315.2410	Pos	M+H	316.2503	6.51	2.65	↑	4.10E-04
5.78	Dodecanoylcarnitine	C19H37NO4	343.2723	Pos	M+H	344.2829	9.71	1.94	↑	1.99E-02
8.25	Palmitoyl-carnitine	C23H45NO4	399.3349	Pos	M+H	400.3441	5.01	1.79	↑	1.08E-03
13.28	Stearidonic Acid ethyl ester	C20H32O2	304.2402	Pos	M+H	305.2495	6.69	2.31	↑	1.66E-02
2.72	Phenylalanine	C9H11NO2	165.0790	Neg	M-H	164.0727	6.12	1.64	↓	2.22E-03
5.14	Glycocholic acid	C26H43NO6	465.3090	Neg	M-H	464.3039	4.64	3.85	↓	9.13E-03
13.05	Docosahexaenoic acid	C22H32O2	328.2424	Neg	M-H	327.2351	6.47	3.52	↑	8.08E-03
14.06	11(Z),14(Z),17(Z)-Eicosatrienoic Acid	C20H34O2	306.2559	Neg	M-H	305.2515	9.55	3.36	↑	1.91E-02
14.74	Oleic acid	C18H34O2	282.2579	Neg	M-H	281.2506	7.06	3.61	↑	3.86E-03
2.98/2.99	Tryptophan	C11H12N2O2	204.0910	Pos/Neg	M+H/M-H	205.0970/203.0837	-0.95/5.50	1.76/1.57	↓	8.52E-06/5.53E-05
7.55/7.57	LPC 18:3	C26H48NO7P	517.3168	Pos/Neg	M+H/M+FA-H	518.3263/562.3151	4.29/0.12	1.60/1.70	↓	2.3E-02/1.84E-02
8.02/8.04	LPE 18:2	C23H44NO7P	477.2855	Pos/Neg	M+H/M-H	478.2956/476.2791	5.93/1.83	1.75/1.73	↓	2.83E-03/5.93E-03
8.13/8.04	LPE 22:6	C27H44NO7P	525.2855	Pos/Neg	M+H/M-H	526.297/524.2789	7.92/1.18	1.83/2.08	↑	3.9E-03/3.59E-03

* For metabolites detected in both positive and negative ion modes, the one with smallest FC was selected for further analysis.
