# Supplementary material for: Evaluation of Untargeted Metabolomic Strategy for the Discovery of Biomarker of Breast Cancer
Source: Front Pharmacol. 2022 May 30;13:894099. doi: 10.3389/fphar.2022.894099 (PMC9189413; doi:10.3389/fphar.2022.894099)
Supplement: Supplementary file 2 [file Image1.pdf]

Supplementary Material

Evaluation of Untargeted Metabolomic Strategy for the Discovery of Biomarker of Breast Cancer

Xujun Ruan [a,1], Yan Wang [c,1], Lirong, Zhou [c], Qiuling Zheng [c] *, Haiping Hao [a] *, Dandan He [b] *

[a] Key Laboratory of Drug Metabolism and Pharmacokinetics, State Key Laboratory of Natural Medicines, China Pharmaceutical University, Tongjiaxiang #24, Nanjing, Jiangsu, 210009, China

[b] Experimental Center of Molecular and Cellular Biology, the Public Laboratory Platform, China Pharmaceutical University, Tongjiaxiang #24, Nanjing, Jiangsu, 210009, China

[c] Department of Pharmaceutical Analysis, College of Pharmacy, China Pharmaceutical University, Tongjiaxiang #24, Nanjing, Jiangsu 210009, China.

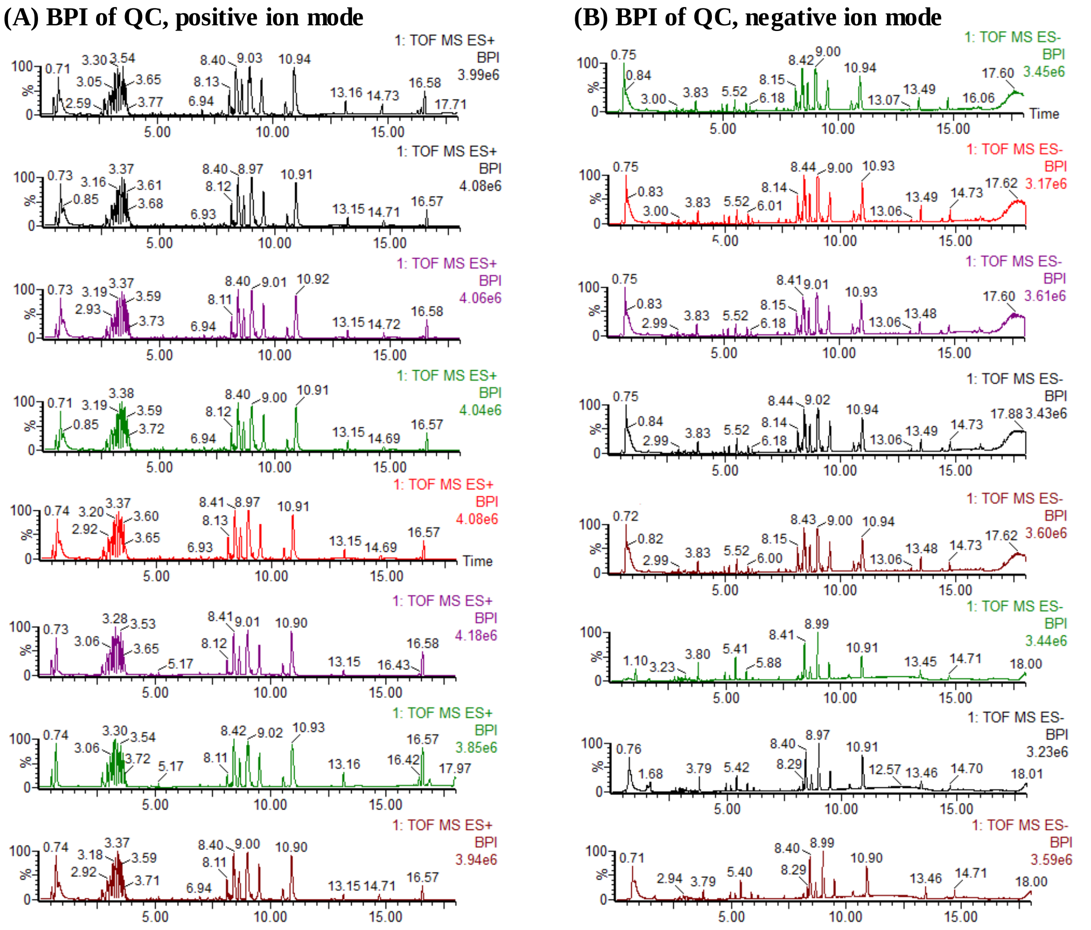

Fig S1. Base peak chromatogram of five successive injections and interval injections of QC samples in (A) the positive ion mode and (B) the negative ion mode.

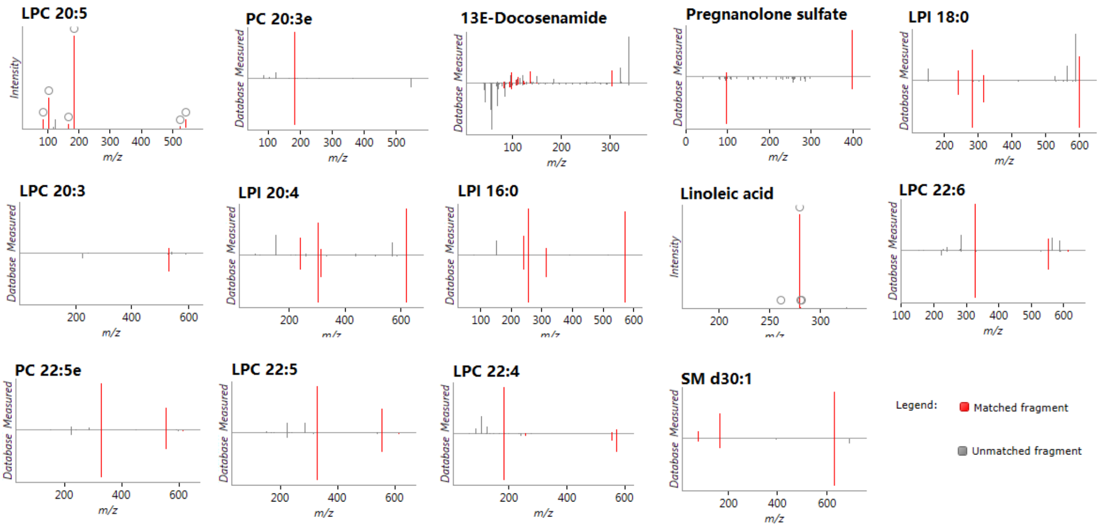

Fig S2. Fragmentation match of differential metabolites between healthy control and breast cancer group based on MS/MS spectra database or fragment simulation

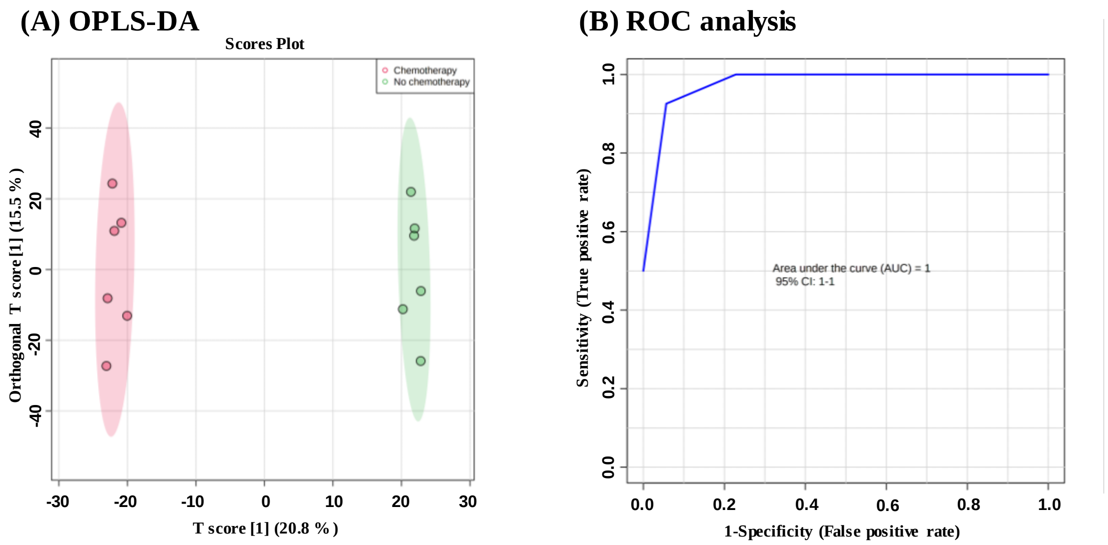

Fig S3. (A) OPLS-DA analysis of samples from breast cancer patients before and after chemotherapy; and (B) ROC analysis of differential metabolites.

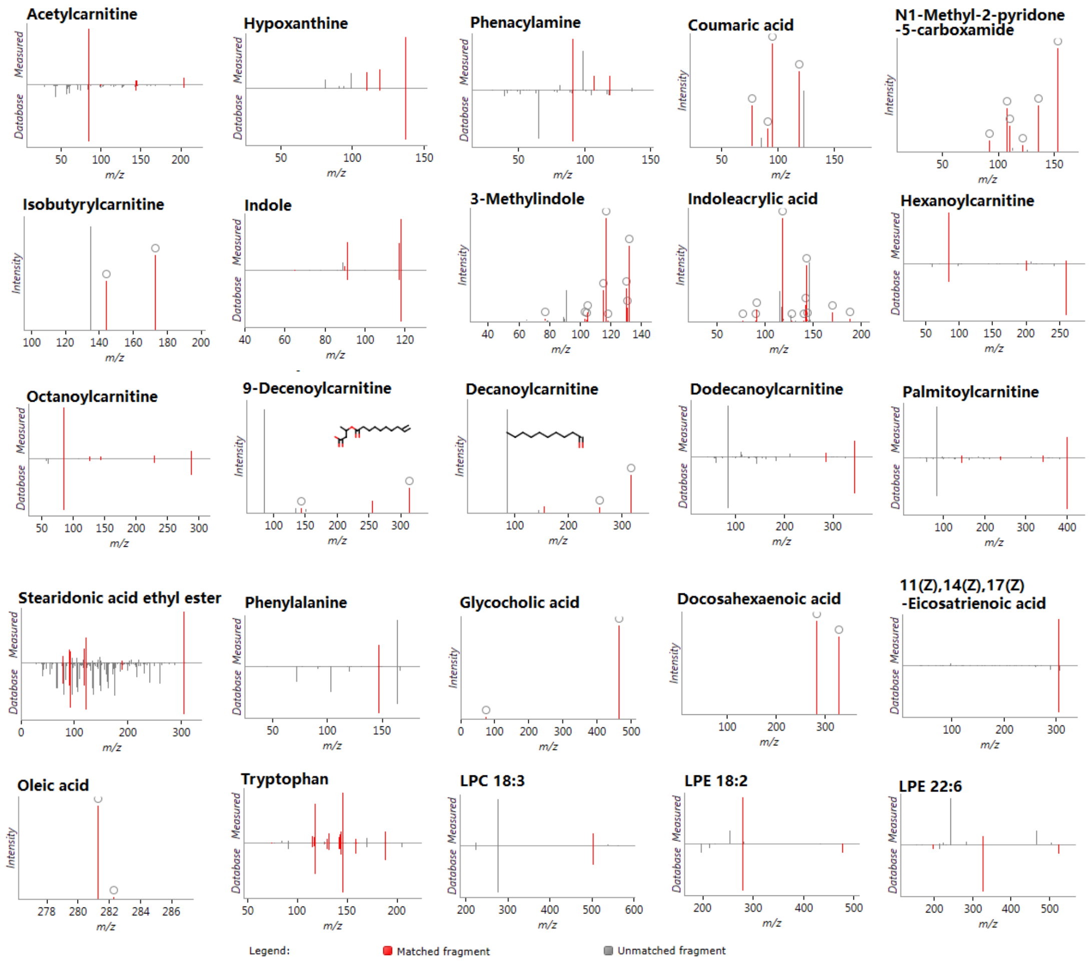

Fig S4. Fragmentation match of differential metabolites of breast cancer before and after chemotherapy based on MS/MS library or fragment simulation.
